# Marine Biogenic Minerals Hold Clues About Changes in Ocean Chemistry and Climate: Some Important Lessons Learned from Studies of Stable and Radioactive Isotopes of Be and Al

**DOI:** 10.1100/tsw.2002.292

**Published:** 2002-05-11

**Authors:** Devendra Lal

**Affiliations:** Scripps Institution of Oceanography, Geosciences Research Division, La Jolla, CA 92093, USA

**Keywords:** beryllium isotopes, aluminum isotopes, marine biogeochemistry, corals

## Abstract

The elements Be and Al exhibit very short residence time in ocean waters, and therefore serve as useful tracers for the study of biogeochemical processes in seawater. A unique feature of these tracers is that nuclear interactions of cosmic rays in the atmosphere produce appreciable amounts of two radioactive isotopes, Be (with a half-life of 1.5 my) and Al (with a half-life of 0.7 my), which are introduced in the hydrosphere, cryosphere, and lithosphere via precipitation. Thus, these elements are labeled by their respective radioactive isotopes, which help quantitative tagging of their biogeochemical cycles. Finally, as we report here, several marine organisms incorporate them in their skeletal shells in certain fixed proportions to their concentrations in the seawater, so that it seems possible to study changes in the ocean chemistry and climate over the past several million years. We summarize here the recent discovery by Dong et al.[9] of significant enrichments of intrinsic Be and Al in marine foraminiferal calcite and coral aragonite, and of Al in opal (radiolarians) and aragonite (corals), which should make it possible to determine Be/Be and Al/Al in oceans in the past. We also summarize their measured Be/Be in foraminiferal calcite in Pacific Ocean cores, which reveal that the concentrations and ratios of the stable and cosmogenic isotopes of Be and Al have varied significantly in the past 30 ky. The implications of these results are discussed.

## INTRODUCTION

Since the pioneering observations by Merrill et al.[1] of the very low concentrations of dissolved $^{9}Be$ in seawater, and those by Raisbeck et al.[2] of the ultra-low concentrations of dissolved $^{10}Be$ in the oceans, a number of measurements have been reported for the concentrations of these isotopes in vertical profiles in the principal oceans[3,4,5]. The recent careful studies of dissolved concentrations of $^{26}Al$ and Al in seawater by Ku et al.[6] have now added another paleo-tracer for studying the marine biogeochemistry of a second particle-active element with a cosmogenic isotope. The studies of Ku et al.[6] revealed that Al is removed 30 times faster than Be from surface ocean waters, and that Al should serve as an ideal paleo-tracer for biological productivity.

The database on distribution of Be and Al isotopes in the oceans, and in marine sediments and accretions, is quite extensive, and it throws considerable light on the marine geochemistry of beryllium as controlled by biology, climate, and aeolian fluxes to the ocean. The advantages of making simultaneous measurements of $^{10}Be$ and $^{9}Be$ on the one hand and of $^{26}Al$ and Al on the other lies in the fact that the radionuclides of these elements are produced by cosmic rays, essentially at a constant rate[7] and independent of climate, whereas the dissolved elemental concentrations of Be and Al depend on factors such as climate, marine biological productivity, and (aeolian) dust flux; the last two factors can be strongly coupled to climatic changes. Changes in the production rate due to high-frequency variations in solar activity are not seen in the ocean. However, geomagnetic field changes would produce appreciable changes in the production rate. A decrease in the geomagnetic field intensity by a factor of 2 would increase the mean global production rate by a factor of 1.3. Furthermore, whereas the absolute Be (or $^{10}Be$) and Al (or $^{26}Al$) concentrations in marine materials may vary with time depending on the geomagnetic field and ocean chemistry and temperature, the $^{10}Be/Be$ and the $^{26}Al/Al$ ratios would be fixed in the proportion they exist in the seawater. The isotope/element ratios, rather than the absolute Be or $^{10}Be$, or Al or $^{26}Al$ concentrations, are therefore a better representation of changes in ocean chemistry, since the absolute concentrations are expected to display larger spatial variability.

We thus see that by using the Be and Al isotopes in conjunction with the elemental concentrations of Be and Al in ocean waters we can learn a great deal about the geographical variations in oceanic productivity and about their geochemistry. We would like to mention here an interesting paper by Peng et al.[8], who have synthesized the global oceanic data on the concentrations of Be and $^{10}Be$. They used this data to obtain detailed insight into the oceanic residence times of Be in different depth intervals in the principal oceans, which are controlled by two main factors: input of Be from the continents via riverine and eolian flux, and local residence time in the seawater column. Since the element Al exhibits shorter residence time in the oceans, and is therefore more ideally suited for studying biological productivity as discussed above, it becomes manifest that a simultaneous study of the paleo-oceanic concentrations of Be, Al, and their isotopes would prove very rewarding for delineating temporal changes in ocean chemistry and biological productivity. The recent studies of Dong et al.[9] seem to offer a potential method for the studies of paleo-concentrations of dissolved Be and Al isotope concentrations in ocean waters, based on observations of diverse marine biogenic minerals in which dissolved Be and Al are incorporated, and therefore it should be possible to study past changes in ocean chemistry and biological productivity.

## RESULTS OF STUDIES OF INTRINSIC CONCENTRATIONS OF BE AND AL IN DIVERSE MARINE MINERALS

To identify suitable archives of the cosmogenic Be and Al isotopic records of seawater in the past, Dong et al.[9] analyzed three principal biogenic materials: foraminiferal calcite, coral

**TABLE 1**
**Measured Intrinsic Concentrations of Be and Al in the Three Biogenic Marine Minerals**

| Biogenic Mineral | Concentration of the Element (ppb)* | | Specific Concentrations (atom/atom) | |
|---|---|---|---|---|
| | **Be** | **Al** | **Be/Ca** | **Al/Ca** |
| Calcite (foraminifera) | ~10 | ~20 | $10^{-7}$ | $10^{-7}$ |
| Coral (aragonite) | 1–2 | 200–800 | (1–2) x $10^{-8}$ | (3–10) x $10^{-8}$ |
| | Be | Al | Be/Si | Al/Si |
| Opal | 50–200 | ~$10^{3}$ ppm | (0.5–1.5) x $10^{-7}$ | ~2 x $10^{-3}$ |

*Unless otherwise specified.

aragonite, and radiolarian opal. Based on the experience gained in measuring the intrinsic concentrations of Be and Al, free from contaminations from any occluded clay and detrital minerals, and by suitable checks on the results, they obtained information on the partitioning of Be and Al in the $CaCO_3$ and $SiO_2$ lattice structures of foraminifera, coral, and opal in the seawater. Their data are summarized in Table 1.

Dong et al.[9] also studied the stable isotopes of Be and Al in all the three biogenic materials, and conducted a study of cosmogenic $^{10}$Be in two foraminiferal calcite-rich box cores; results are plotted in Fig. 1. The calcite samples were from two neighboring box cores from the equatorial Pacific Ocean taken during the Eurydice expedition (Lat. 0°S, 1°N, Long. 161°E, and Lat. 0°52'N, Long. 161°E; water depth = 3,420–3,430 m). Opal samples were from the Ocean Drilling Program, Leg 177, Hole 1091A (47°5.681'S, 5°55.120'E), water depth 4,360.5 mbs, from the Holocene section of the core. The coral sample was collected in 1990 under 10 m of water, near the town of Padang Bali on the southeast coast of Bali. Calcite and opal fragments were separated from the cores, cleansed of detrital clay, and analyzed for $^{10}$Be following standard procedures as developed for analysis using the accelerator mass spectrometry.

## DISCUSSION

To judge the suitability of the selected biogenic materials as archives for dissolved Al and Be in seawater, and thus as proxies for trace nutrients derived from the dissolution of windblown detrital minerals, Dong et al.[9] compared the intrinsic elemental Be and Al concentration data obtained from core tops (0–5 cm) in modern samples with estimates of the present average elemental concentrations in seawater. Seawater values adopted for the comparison are the average values reported in the literature for modern Pacific Ocean surface waters in units of moles per liter (M): Be = 10 pM; Al = 2 nM; Ca = 10 mM; and Si = 10 μmol. A comparison of these average modern-day seawater values with those in the table above, based on analyses of biogenic archives, revealed the following:

1. In foraminiferal calcite, *relative to Ca,* Be is enriched by a factor of ~100 over that in seawater and Al is depleted by a factor of about 3. Both Be and Al are included in appreciable concentrations in the calcite matrix, as is also seen by the positive correlation between Be and Al (Figs. 1b and c).

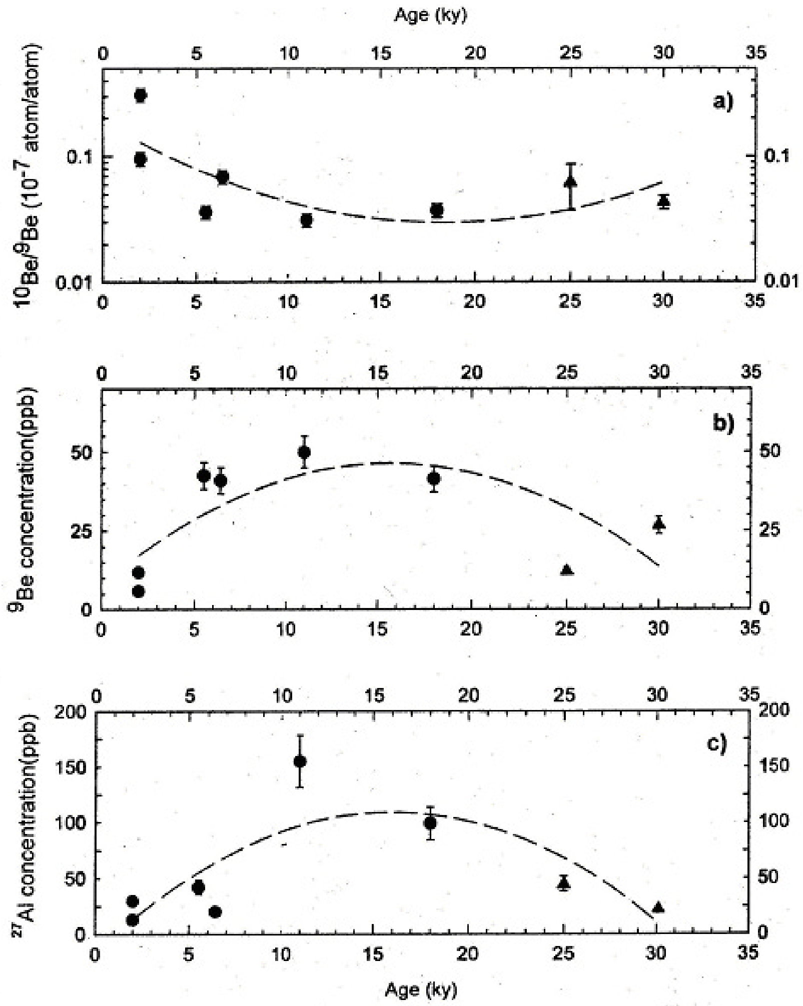

**FIGURE 1.** Measured concentrations of intrinsic Be, Al, and $^{10}$Be concentrations in foraminiferal calcite from the equatorial Pacific Ocean box cores ERDC 125 (circles) and ERDC 135 (triangles).

2. In coral aragonite, *relative to Ca,* Be is enriched by a factor of ~20 over that in seawater and Al is enriched by a factor of ~10.
3. In radiolarian opal, *relative to Si,* Be is neither enriched nor depleted compared to seawater, but Al is enriched by a factor of about 10, as in corals.
4. The $^{10}$Be/Be ratios of present-day surface ocean waters[3,4] are comparable to the $^{10}$Be/Be ratios in foraminiferal calcite.
5. Figures 1a, b, and c, respectively, show plots of $^{10}$Be/$^{9}$Be ratios, as well as Be and Al concentrations with their uncertainties for the ERDC box cores. The absolute concentrations of $^{10}$Be, Be, and Al values in Fig. 1 show considerable variation, by an order of magnitude, at 30 ky BP, illustrating that the dissolved concentrations of continentally derived Be and Al change significantly with time, thereby providing a potentially sensitive chemical indicator of global change.

These results suggest that foraminiferal calcite and coral aragonite are excellent repositories for paleoconcentrations of seawater-dissolved Be. For dissolved Al, coral aragonite and diatom opal are both satisfactory. Of the three, coral occupies the middle range for suitability for determining both Be and Al concentrations. Calcite, however, is not useful for determining concentrations of dissolved Al.

As shown in Fig. 1, there is an inverse relationship between $^{10}Be/^{9}Be$ and the intrinsic trace element concentration of $^{9}Be$. This study also shows a similar relationship for $^{10}Be/^{9}Be$ and $^{27}Al$ concentrations. Unfortunately, in our data set there are large time gaps between points. As a result, the actual shape of oscillations and the fine structure of the curves in Figs. 1a, b, and c have not yet been determined. Nevertheless, fitting the data with a second-order polynomial yields a crude minimum in $^{10}Be/^{9}Be$ ratios and a maximum in $^{9}Be$ and $^{27}Al$ concentrations at 15–20 ky BP. These data structures are in reasonable agreement with the end of the last glacial maximum (~20 ky BP) when the global climate was significantly cooler than today. Low $^{10}Be/Be$ ratios should be expected during times of intense glaciation owing to increased wind forcing that is generally tied to cooler climatic conditions. This wind forcing increases the continental dust flux to the oceans, resulting in the dilution of cosmic ray–generated $^{10}Be$ with Be. We may also note here that the observed low ratios also correspond with observed increases in paleoproductivity[10].

Although similar isotopic ratios have yet to be measured for Al or Be in the other marine biogenic materials studied (coral aragonite and radiolarian opal), the presence of cosmogenic $^{9}Be$ and $^{27}Al$ and reasonable concentrations of total Be and Al in these materials (as summarized in Table 1) would suggest that similar data sets from these materials can also be generated.

## CONCLUSIONS

The results of Dong et al.[9] clearly establish the dramatic possibility of studying the paleogeochemistry of seawater with stable and cosmogenic Be and Al isotopes using biogenic calcite, aragonite, and opal. An important result of this work is that with the present day sensitivity of $10^{6}$ atoms for $^{10}Be$ and $^{26}Al$, and with dissolved $^{10}Be/Be$ and $^{26}Al/Al$ ratios in the range of $10^{-8}$–$10^{-7}$ and $10^{-13}$–$10^{-14}$, respectively measurable amounts of $^{10}Be$ can now be detected in anywhere from less than a single gram to a few grams of modern calcite or opal. Similarly, $^{26}Al$ can be detected in 1–5 g of modern opal. This high sensitivity makes it possible to now carry out paleoceanographic studies of Be and Al in a variety of marine sediments.

This study also indicates that the isotopic ratios of Be and Al can shed valuable light on how and why ocean productivity has varied in the past — a key to understanding climate controls and burial of organic matter in the oceans. Full-scale validation of our admittedly sparse data set has the potential to open up new avenues for quantifying biological productivity variations and their ties to continental dust flux and its effect on boosting the dissolved nutrient content of seawater. This, in turn, can be related quantitatively to organic matter preservation in sediments and, thus, improve our understanding of the carbon cycle and its change over time A bonus of this technique is that, because Be and Al ratios are tied to wind forcing, they should also serve to couple changes in trade wind strength, and thus paleoclimate and ocean paleoproductivity. This new window into the oceanic past will hopefully establish new links to cause-effect scenarios, e.g., climate and ocean circulation, atmospheric carbon dioxide concentrations, climate, etc.

## ACKNOWLEDGEMENTS

We are grateful to Mr. Myron Eichen for the generous support for these studies.
